# Mechanism of LINC00958 in ferroptosis of breast cancer through the SRSF1/GPX4 axis

**DOI:** 10.1186/s41065-025-00469-6

**Published:** 2025-06-19

**Authors:** Shan Wang, Xinlu Liu, Xiaoli Hou, Wei Sun, Jiajie Chen, Yasen Cao, Hong Cheng

**Affiliations:** 1 Jiangsu Key Laboratory of Experimental & Translational Non-coding RNA Research, Institute of Translational Medicine, Yangzhou University Medical College, Yangzhou University, Room 223, Building 6, No.136 Jiangyang Middle Road, Yangzhou, 225000 Jiangsu China; 2 The Second People’s Hospital of Lianyungang City (Cancer Hospital of Lianyungang), Lianyungang, 222002 China; 3 Department of Medical Science, Yangzhou Polytechnic College, Yangzhou, 225000 China

**Keywords:** LINC00958, Breast cancer, SRSF1, GPX4, Ferroptosis, RNA-binding protein

## Abstract

**Background:**

Breast cancer (BC) is the most common cancer among women. Ferroptosis is a novel iron-dependent form of cell death and affects cancer development. This study was conducted to explore the role of long intergenic non-protein coding RNA958 (LINC00958) in the ferroptosis of BC cells.

**Methods:**

The expression levels of LINC00958 and glutathione peroxidase 4 (GPX4) in BC cell lines were first determined. After the interference of LINC00958, cell proliferation was assessed by cell counting kit-8 and colony formation assays, and ferroptosis was tested by measurements of ferric ion content, reactive oxygen species (ROS), glutathione (GSH), and acyl-CoA synthetase long-chain family member 4 (ACSL4). The binding of serine/arginine splicing factor 1 (SRSF1) to LINC00958/GPX4 was analyzed by RNA immunoprecipitation. GPX4 mRNA stability was determined after actinomycin D treatment. Rescue experiments were conducted to test the role of GPX4 in ferroptosis. Finally, mouse transplantation tumors were designed to verify the role of LINC00958.

**Results:**

LINC00958 was highly-expressed in BC cells. LINC00958 downregulation inhibited proliferation and promoted ferroptosis, while LINC00958 overexpression promoted proliferation and inhibited ferroptosis. LINC00958 directly bound to SRSF1, increased the occupancy of SRSF1 on GPX4 mRNA, and augmented the mRNA stability of GPX4. GPX4 overexpression neutralized the promotive role of LINC00958 downregulation in ferroptosis of BC cells.

**Conclusion:**

Binding of LINC00958 to SRSF1 increases the occupancy of SRSF1 on GPX4 mRNA and the mRNA stability and expression of GPX4, thereby inhibiting ferroptosis of BC cells.

**Supplementary Information:**

The online version contains supplementary material available at 10.1186/s41065-025-00469-6.

## Introduction

Breast cancer (BC) is a complex and common malignant tumor that primarily affects women, typically originating from cells within the breast ducts [1]. Globally, BC is the leading cause of cancer deaths, with 685,000 deaths in 2020 [2]. Age, family history, reproductive factors, estrogen, and unhealthy lifestyle choices are risk factors for BC [3]. Hormonal therapy, surgical management, and chemotherapy drugs are commonly used treatments, but resistance and side effects still exist and may lead to poor efficacy [4]. Ferroptosis is a form of cell death where ferroptosis inducers affect glutathione peroxidase to decrease the cell’s antioxidant capacity and the accumulation of lipid-reactive oxygen species (ROS), which promotes lipid peroxidation and ultimately triggers cell death [5]. Inducing ferroptosis in BC cells may be a promising therapeutic approach [6].

Long non-coding RNAs (lncRNAs) are non-protein coding RNA transcripts longer than 200 nucleotides and play important roles in cellular processes [7]. Long intergenic non-protein coding RNA 958 (LINC00958) is highly expressed in various malignancies and is considered a novel target for tumor therapy due to its role in enhancing resistance to radiotherapy and chemotherapy in tumor cells [8]. Moreover, it is widely reported that LINC00958 functions as an oncogene in multiple types of cancers, including bladder cancer [9], colon cancer [10], endometrial cancer [11], and cervical cancer [12]. More importantly, LINC00958 is significantly overexpressed in BC tissues and cells [13]. Although lncRNAs are pivotal modulators of ferroptosis in cancer cells [14], there are currently no studies reporting the involvement of LINC00958 in BC through ferroptosis.

Glutathione peroxidase 4 (GPX4) is a member of the GPX family that uses glutathione (GSH) as a cofactor to reduce peroxides to their corresponding alcohols, thereby maintaining lipid homeostasis and preventing the accumulation of toxic lipid ROS, ultimately limiting ferroptosis [15]. Thus, GPX4 has a protective role in shielding cells from ferroptosis triggered by oxidative stress [16]. Currently, targeting GPX4 for inducing ferroptosis and promoting cancer cell death has been proven to be a new strategy for cancer treatment [17]. In BC, ferroptosis mediated by GPX4 inactivation can alleviate drug resistance, inhibit tumor growth, and enhance antitumor immunity [18, 19]. A previous study has shown that abnormally expressed lncRNAs can regulate GPX4 protein levels, thereby affecting the progression of BC [20]. Our research further revealed the role of LINC00958/GPX4 in BC through ferroptosis.

Within this investigation, we probed the function of LINC00958 in ferroptosis of BC cells and elucidated its potential mechanism in BC, providing new theoretical support for BC treatment.

## Materials and methods

### Ethics statement

Animal experiments were conducted under the guidance of Animal Care and Use Committee of Yangzhou University Medical College. Animal studies were performed in compliance with the ARRIVE guidelines. All procedures involving the use, care, and handling of animals were complied with the National Institutes of Health Guide for the Care and Use of Laboratory Animals [21].

### Cell culture and transfection

The human BC cell lines MDA-MB-157 (https://www.cellosaurus.org/CVCL_0618; ER-/PR-/HER2-/Low TNBC subtype), MDA-MB-468 (https://www.cellosaurus.org/CVCL_0419; ER⁻/PR⁻/HER2⁻ TNBC subtype), MDA-MB-231 (https://www.cellosaurus.org/CVCL_0062; ER⁻/PR⁻/HER2⁻ TNBC subtype) and the normal human breast epithelial cell line MCF10A (https://www.cellosaurus.org/CVCL_0598; ER⁺/PR⁺/HER2⁻ epithelial cell) were sourced from the American Type Culture Collection (ATCC, Manassas, VA, USA). The cells were cultured in Dulbecco’s modified Eagle’s medium (Invitrogen, Gaithersburg, MD, USA) containing 10% fetal bovine serum (Invitrogen) and 1% penicillin-streptomycin at 37 °C with 5% CO_2_.

Cells were used for transfection when they reached 80% confluence. Transfections were carried out using Lipofectamine 3000 reagent, following the manufacturer’s guidelines (Thermo Fisher Scientific, Waltham, MA, USA) to introduce LINC00958/GPX4 pcDNA3.1, empty pcDNA3.1, LINC00958/GPX4/SRSF1 siRNA, and NC siRNA (RiboBio, Guangzhou, China) into the cells. MDA-MB-231 cells were infected with lentivirus carrying LINC00958 knockdown (sh-LINC00958) and negative control lentivirus (sh-NC) (GenePharma, Shanghai, China) at a multiplicity of infection of 20. siRNA sequences are showed in Supplementary Table 1.

### Cell counting kit-8 (CCK-8) assay

Cells were seeded in a 96-well plate (1 × 10^4^ cells/well) for 24 h. After transfection of 48 h, cells were experienced 2-hour incubation with 10 µL of CCK-8 solution, and the optical density (OD) value of each well was measured at 450 nm using a microplate reader (BioTek, Winooski, Vermont, USA).

### Iron, ROS, and GSH detection

Iron content in cells was assessed utilizing an iron assay kit (Sigma-Aldrich, St. Louis, USA) following the manufacturer’s protocol. Briefly, 2 × 10^6^ cells were digested with trypsin, and iron assay buffer was added. After centrifugation, the supernatant was collected and transferred to a 96-well plate (100 µL/well). Then, each well received 5 µL of iron assay buffer, followed by mixing and incubation in the dark at 25 °C for 30 min. Finally, 100 µL of iron probe was added to each well incubated in the dark at 25 °C for 60 min, and the OD value of each well was measured at 593 nm using a microplate reader. Tissue iron content was determined following the instructions.

Cells were incubated with 10 µM 2′,7′-Dichlorofluorescin diacetate probe (Beyotime, Shanghai, China) for 25 min at 37 °C. After incubation, cells were washed twice with phosphate-buffered saline and fluorescence was detected using a BX53M fluorescence microscope. Fluorescence intensity analysis was performed using ImageJ (version 1.50; National Institutes of Health, Bethesda, USA). ROS content in tissues was measured according to the instructions.

GSH levels were detected using a GSH assay kit (S0052, Beyotime). Briefly, cells were seeded in 6-well plates for 24 h. They were then harvested and resuspended in the reagents as per the provided guidelines. The absorbance of the product was measured at 412 nm using a UV spectrophotometer (Thermo Fisher Scientific). GSH content in tissues was measured following the instructions.

### Nuclear-cytoplasmic fractionation assay

Cells were seeded at a density of 0.5 × 10^5^ cells/well in a 12-well plate containing cell coverslips and incubated overnight at 37 °C in a 5% CO_2_ incubator. After washing, cells were fixed in 4% paraformaldehyde. The RNA FISH kit (GenePharma) was used in conjunction with a specific probe mixture. The target probe for LINC00958 was purchased from RiboBio. Cells were then counterstained with 4’,6-diamidino-2-phenylindole working solution. After washing, clean slides were treated with an anti-quench agent and mounted onto the cellular side of the slides. Finally, images were captured using a confocal laser scanning microscope (Zeiss, Oberkochen, Germany).

To ascertain the proportion of LINC00958 in the nuclear and cytoplasmic compartments, we employed the NE-PER™ Nuclear and Cytoplasmic Extraction Kit (Thermo Fisher Scientific). LINC00958 expression in the nuclear and cytoplasmic fractions was determined by reverse transcription quantitative polymerase chain reaction (RT-qPCR). U6 was used as a nuclear positive control, and glyceraldehyde-3-phosphate dehydrogenase (GAPDH) was used as a cytoplasmic positive control.

### Bioinformatics

The subcellular localization of LINC00958 was predicted using the lncATLAS database (https://lncatlas.crg.eu/?tdsourcetag=s_pcqq_aiomsg) [22]. The binding interactions of LINC00958, SRSF1, and GPX4 were predicted using the RNAInter database (http://www.rna-society.org/rnainter/) [23] and the StarBase database (https://rnasysu.com/encori/index.php) [24]. The binding probability of SRSF1 with LINC00958 and GPX4 was predicted using the RPISeq database (http://pridb.gdcb.iastate.edu/RPISeq/) [25].

### RNA pull-down assay

The full-length LINC00958 was synthesized in vitro, labeled with biotin (Sangon, Shanghai, China), incubated with cell lysate for 3 h, and added by streptavidin magnetic beads (Thermo Fisher Scientific) for 0.5 h-incubation. Pulled-down proteins were analyzed by Western blot. Immunoglobulin G (IgG) (ab172730, Abcam) was used as the negative control.

### RNA Immunoprecipitation (RIP) assay

RIP assays were conducted using the Magna RIP kit (Millipore, Billerica, MA, USA). Briefly, magnetic beads were incubated with SRSF1 antibody (ab38017, Abcam, Cambridge, MA, USA) or IgG negative control antibody (ab172730, Abcam). Subsequently, cells were lysed and incubated with antibody-coated beads. Co-precipitated RNA was then extracted using TRIzol reagent (Takara, Dalian, China) and detected by RT-qPCR.

### mRNA stability assay

To assess RNA stability, cells were exposed to actinomycin D (1 µg/mL), and RNA was harvested at various time points for RT-qPCR analysis.

### Xenograft experiment in nude mice

Six-week-old male BALB/c nude mice were purchased from Hunan SJA Laboratory Animal Co., Ltd (Changsha, Hunan, China). MDA-MB-231 cells (2 × 10^7^ cells/mL) were injected subcutaneously into the right flank of the mice. Tumor size was measured every four days. On day 28 post-xenograft, mice were euthanized via intraperitoneal injection of 150 mg/kg sodium pentobarbital. Tumor volume was calculated using the formula length × width^2^/2. Excised tumor tissues were weighed for subsequent analyses.

### RT-qPCR

Total RNA was isolated using TRIzol reagent (Takara) according to the manufacturer’s protocol. The first-strand complementary DNA (cDNA) was synthesized using the SuperScript First-Strand Synthesis System (Invitrogen). Real-time PCR was performed using the FastStart Universal SYBR Green Master Mix (Roche) on the ABI 7500 Fast Real-Time PCR System (Applied Biosystems, Waltham, CA, USA). GAPDH served as an internal reference. Relative expression levels were calculated using the 2^−ΔΔCt^ method [26]. Primers are listed in Table 1.

**Table 1:** PCR primer sequences

Genes	Sequences (5’-3’)
LINC00958	F: GATTCATGCTTCGCACCCCA
	R: CCTCAGAGGGCTGTTCTCCT
GPX4	F: CAAGGGCATCCTGGGAAATG
	R: TCTTGTCGATGAGGAACTGTG
SRSF1	F: GGTTGTCTCTGGACTGCCTC
	R: ACAAACTCCACGACACCAGT
GAPDH	F: GATGCTGGCGCTGAGTACG
	R: GCTAAGCAGTTGGTGGTGC

### Western blot

Cell lysates were prepared using a protein extraction reagent (Thermo Fisher Scientific), and protein concentrations were determined using the bicinchoninic acid (BCA) protein assay kit (Thermo Fisher Scientific). Equal amounts (20 µg) of protein samples were denatured and separated using 10% sodium dodecyl sulfate polyacrylamide gel electrophoresis. Proteins were then transferred onto polyvinylidene fluoride membranes (Immobilon-P; Merck Millipore, Darmstadt, Germany). The membranes were blocked with 3% bovine serum albumin at 25 °C for 1 h and washed three times with Tris-buffered saline with Tween 20 (TBST). Next, the membranes were incubated overnight at 4 °C with the following primary antibodies: ACSL4 (ab155282, 1:10,000), GPX4 (ab125066, 1:1,000), SRSF1 (ab133689, 1:1,000), and β-actin (ab5694, 1:1,000). After TBST washing, the membranes were incubated with a secondary antibody (ab205718, 1:2,000) for 1 h at 25 °C. The membranes visualization was performed using an enhanced chemiluminescence kit (Thermo Fisher Scientific), and relative band intensities were quantified by employing ImageJ software (version 1.50). β-actin was used as an endogenous control. All antibodies were obtained from Abcam. The relevant data and images are shown in Supplementary Tables 2 and the Supplementary file.

### Statistical analyses

All data were analyzed and plotted using SPSS 21.0 statistical software (IBM SPSS Statistics, Armonk, NY, USA) and GraphPad Prism 8.0 software (GraphPad Software Inc., San Diego, CA, USA). Normality and homogeneity of variance tests were performed first, confirming that the data met the criteria for normal distribution and homogeneity of variance. Comparisons between two groups were analyzed by *t*-test. Comparisons among multiple groups were analyzed by one-way or two-way analysis of variance (ANOVA), followed by Tukey’s multiple comparisons test. *p*-values were two-tailed tests, with *p* < 0.05 indicating statistical significance and *p* < 0.01 indicating highly significant differences.

## Results

### LINC00958 expression is increased in BC cells and inhibits ferroptosis

**Fig. 1:**
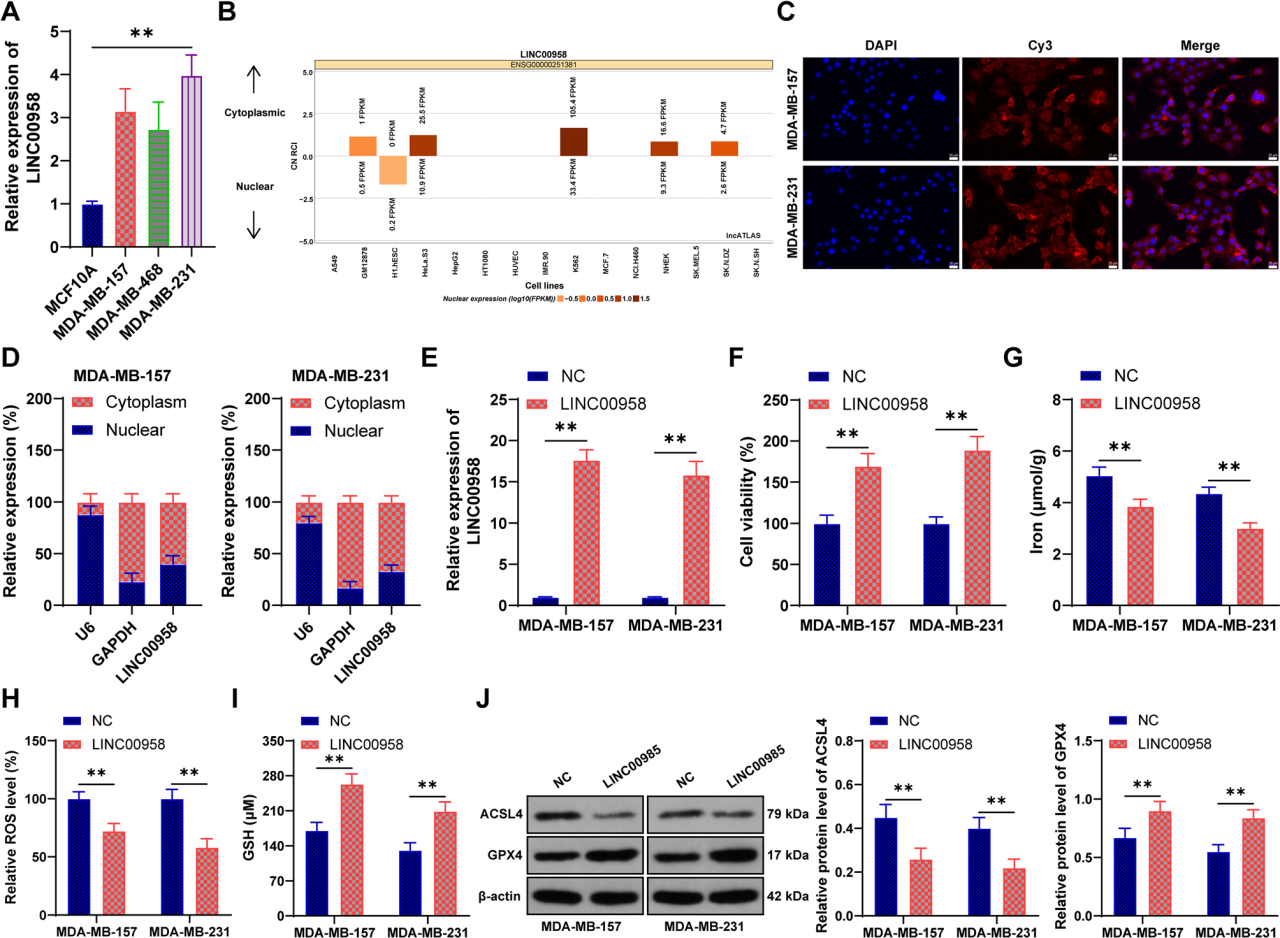
LINC00958 expression is increased in BC cells and inhibits ferroptosis. **A**: RT-qPCR was used to detect the expression of LINC00958 in cell lines. **B**: The lncATLAS database was used to predict the subcellular localization of LINC00958. **C**-**D**: RNA FISH and nuclear-cytoplasmic fractionation experiments were used to verify the localization of LINC00958 in BC cells. LINC00958 pcDNA3.1 (LINC00958) was transfected into cells, with transfection of empty pcDNA3.1 (NC) as the negative control. **E**: RT-qPCR was used to detect the expression of LINC00958 in the cells. **F**: CCK-8 assay was used to measure the viability of BC cells. **G**: Iron content in the cells. **H**: ROS levels in the cells. **I**: GSH levels in the cells. **J**: Western blot was used to detect the protein expression of ACSL4 and GPX4. Experiments were independently repeated three times, and data are presented as mean ± standard deviation. One-way ANOVA was used for comparisons among multiple groups in panel **A**, while two-way ANOVA was used for comparisons among multiple groups in panels **E**-**J**, followed by Tukey’s multiple comparisons test. ** *p* < 0.01

Compared to normal breast epithelial cells (MCF10A), LINC00958 expression was significantly increased in human BC cell lines (*p* < 0.05, Fig. 1A) and mainly localized in the cytoplasm of BC cells (Figs. 1B-D). We selected MDA-MB-157 and MDA-MB-231 cells, which have relatively high expression of LINC00958, for the subsequent experiments. Upregulation of LINC00958 in MDA-MB-157 and MDA-MB-231 cells (*p* < 0.05, Fig. 1E) significantly promoted cell viability (*p* < 0.05, Fig. 1F). Given that the role of LINC00958 in ferroptosis in BC remains unclear, we further examined ferroptosis-related indicators. Overexpression of LINC00958 led to remarkable decreases in iron content and ROS levels and an increase in GSH levels (*p* < 0.05, Figs. 1G-I). Additionally, overexpression of LINC00958 inhibited the protein expression of ACSL4 and promoted the protein expression of GPX4 (*p* < 0.05, Fig. 1J). These results suggest that LINC00958 is primarily located in the cytoplasm, upregulated in BC, and inhibits cellular ferroptosis.

### Downregulation of LINC00958 promotes ferroptosis in BC cells

**Fig. 2:**
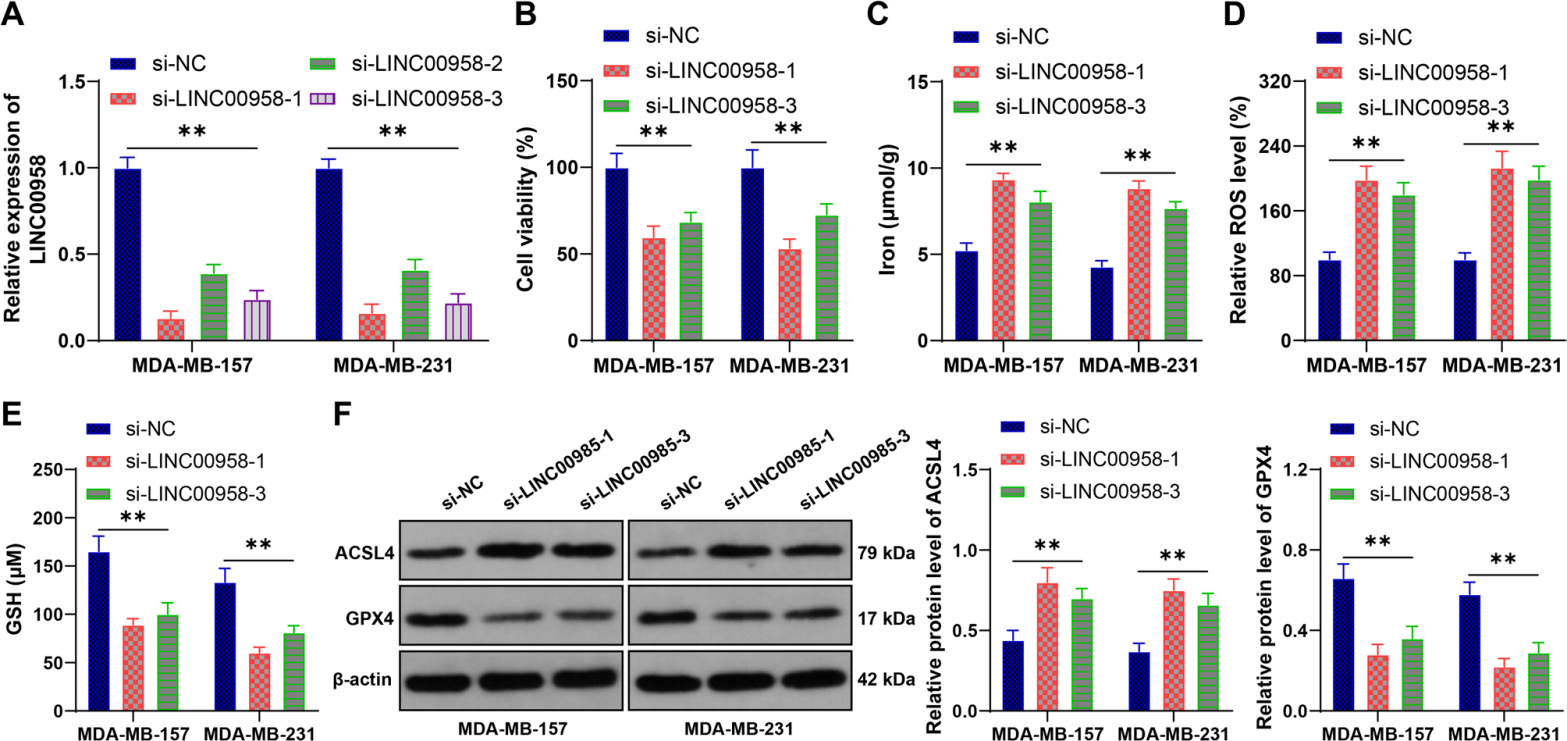
Downregulation of LINC00958 promotes ferroptosis in BC cells. LINC00958 siRNA (si-LINC00958) was transfected into cells, with transfection of NC siRNA (si-NC) as the negative control. **A**: RT-qPCR was used to detect the expression of LINC00958 in the cells. **B**: CCK-8 assay was used to measure the viability of BC cells. **C**: Iron content in the cells. **D**: ROS levels in the cells. **E**: GSH levels in the cells. **F**: Western blot was used to detect the protein expression of ACSL4 and GPX4. Experiments were independently repeated three times, and data are presented as mean ± standard deviation. Comparisons among multiple groups in panels **A**-**F** were analyzed using two-way ANOVA, followed by Tukey’s multiple comparisons test. ** *p* < 0.01

Subsequently, we downregulated LINC00958 expression in MDA-MB-157 and MDA-MB-231 cells (*p* < 0.05, Fig. 2A) and selected two siRNAs with better transfection efficiency for further testing. We found that downregulation of LINC00958 significantly decreased cell viability (*p* < 0.05, Fig. 2B), increased iron content and ROS levels, and decreased GSH levels (*p* < 0.05, Figs. 2C-E). Additionally, LINC00958 downregulation promoted ACSL4 expression and inhibited GPX4 expression (*p* < 0.05, Fig. 2F). These findings suggest that downregulation of LINC00958 promotes ferroptosis in BC cells.

### LINC00958 directly binds to SRSF1 to increase GPX4 mRNA stability

**Fig. 3:**
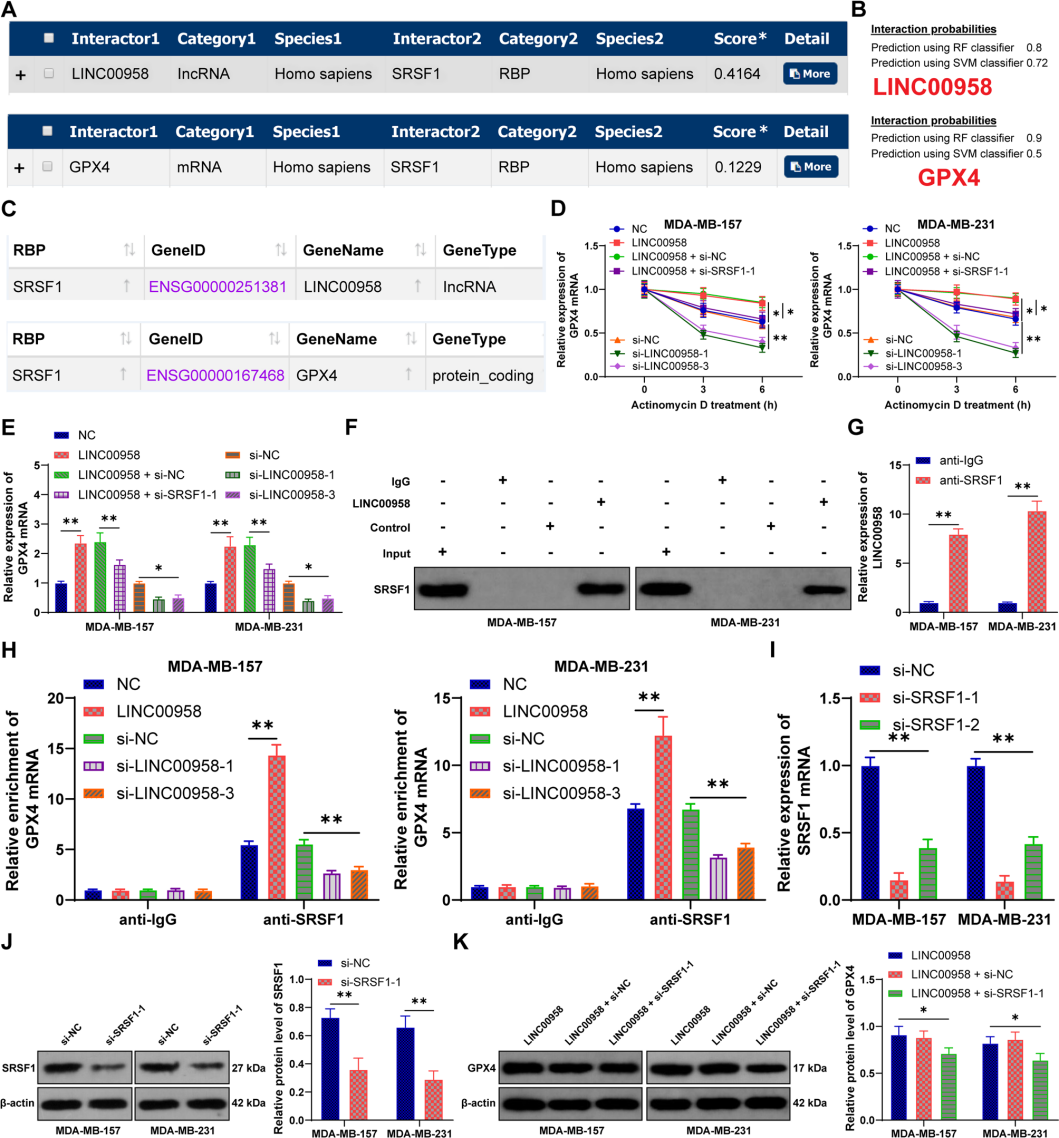
LINC00958 directly binds to SRSF1 to increase GPX4 mRNA stability. **A-C**: Analysis of the binding relationship between SRSF1, LINC00958, and GPX4 using the RNAInter, RPISeq, and StarBase databases. **D**: RT-qPCR was used for GPX4 expression in cells after treatment with actinomycin D. **E**: RT-qPCR was used for GPX4 expression in cells. **F-G**: RNA pull-down and RIP was used for analyzing the binding relationship between SRSF1 and LINC00958. **H**: RIP was used for the binding relationship between SRSF1 and GPX4. **I-J**: RT-qPCR and Western blot were used for SRSF1 protein expression after transfection with si-SRSF1. **K**: Western blot was used for GPX4 protein expression. Experiments were independently repeated three times, and data are presented as mean ± standard deviation. One-way ANOVA was used for comparisons among multiple groups in panel **E**, while two-way ANOVA was used for comparisons among multiple groups in panels **D**, **G**-**K**, followed by Tukey’s multiple comparisons test. * p <0.05, ** p <0.01

Research indicated that lncRNAs block ferroptosis by increasing GPX4 [27]. It remains unclear whether LINC00958 affects ferroptosis in BC cells by enhancing GPX4 expression. Predictions from the RNAInter database identified SRSF1 as an RNA-binding protein with the highest probability of binding to LINC00958, and SRSF1 could also bind to GPX4 (Fig. 3A). Their relationships were validated using the RPISeq and StarBase databases (Figs. 3B-C). The half-life of GPX4 mRNA was slightly increased after upregulating LINC00958, whereas downregulating LINC00958 significantly shortened the half-life of GPX4 mRNA (*p* < 0.05 Fig. 3D). Changes in GPX4 mRNA levels were consistent with its half-life (*p* < 0.05, Fig. 3E). RNA pull-down and RIP results showed that LINC00958 interacted with SRSF1 (*p* < 0.05, Figs. 3F-G). Additionally, changes in LINC00958 expression altered the binding of SRSF1 to GPX4 mRNA in cells (*p* < 0.05, Fig. 3H). Knockdown of SRSF1 (*p* < 0.05, Figs. 3I-J) significantly reduced the half-life of GPX4 mRNA (*p* < 0.05, Fig. 3D) and GPX4 expression (*p* < 0.05, Fig. 3E, K). These findings suggest that LINC00958 increases GPX4 mRNA stability by binding to SRSF1.

### LINC00958 inhibits ferroptosis by increasing GPX4 expression

**Fig. 4:**
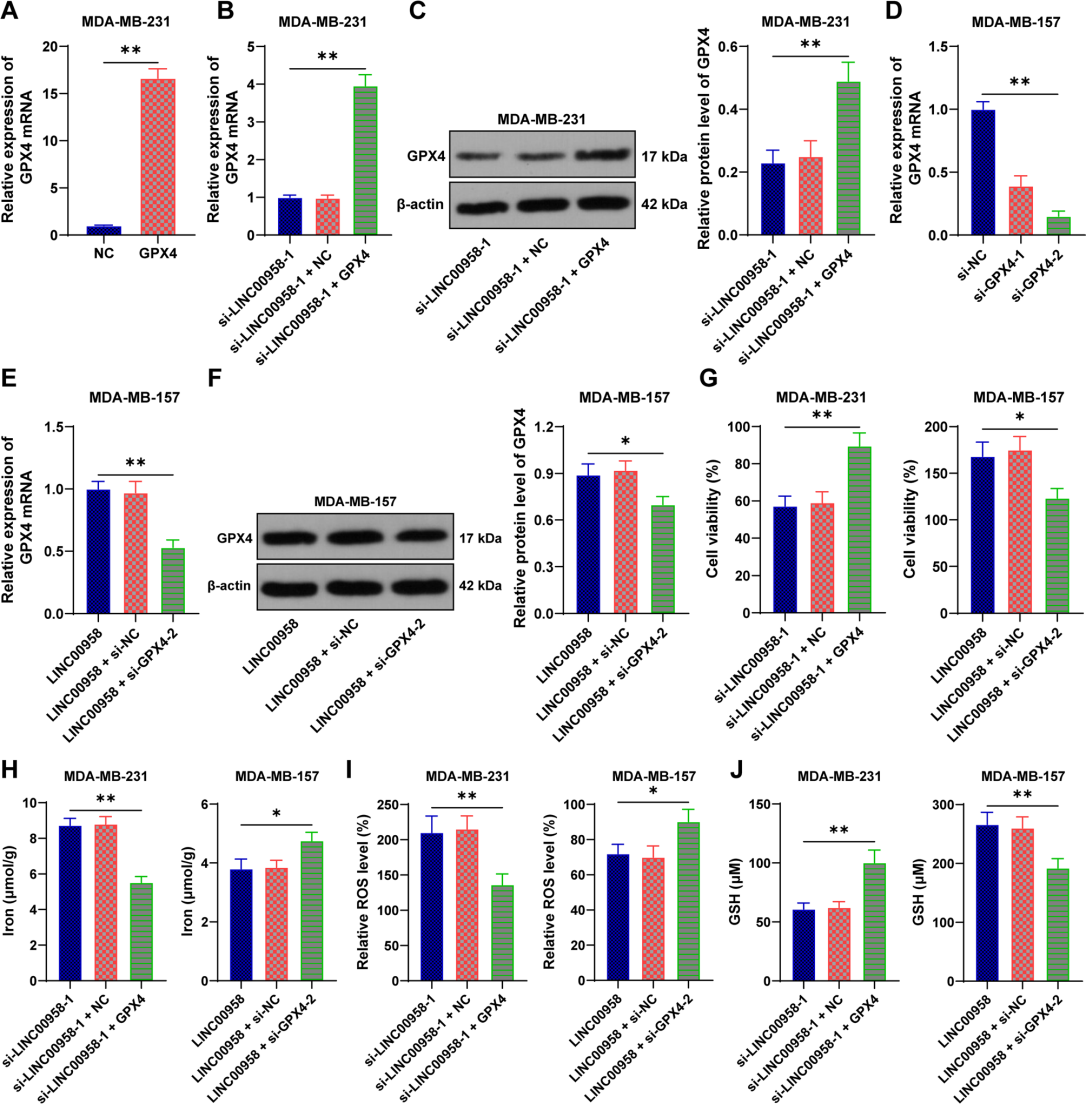
LINC00958 inhibits ferroptosis by increasing GPX4 expression. GPX4 pcDNA3.1 (GPX4) was transfected into MDA-MB-231 cells and co-treated with LINC00958, with transfection of NC as the negative control. GPX4 siRNA (si-GPX4) was transfected into MDA-MB-157 cells and co-treated with si-LINC00958-1, with transfection of si-NC as the negative control. **A-F**: RT-qPCR and Western blot were used to detect the expression of GPX4 in the cells. **G**: CCK-8 assay was used to measure the viability of BC cells. **H**: Iron content in the cells. **I**: ROS levels in the cells. **J**: GSH levels in the cells. Experiments were independently repeated three times, and data are presented as mean ± standard deviation. Comparisons between two groups in panel A were analyzed using t-test, while comparisons among multiple groups in panels B-J were analyzed using one-way ANOVA, followed by Tukey’s multiple comparisons test. * p <0.05, ** p <0.01

Subsequently, we upregulated GPX4 expression in MDA-MB-231 cells with poorly expressed LINC00958 (*p* < 0.05, Figs. 4A-C) and downregulated GPX4 expression in MDA-MB-157 cells with overexpressed LINC00958 (*p* < 0.05, Figs. 4D-F) to conduct combined experiments to verify that LINC00958 affects ferroptosis in BC cells through GPX4. Compared to the group with LINC00958 alone knocked down, the combined experimental group had increased cell viability, decreased iron content and ROS levels, and elevated GSH levels (*p* < 0.05, Figs. 4G-J). Conversely, compared to the group with LINC00958 overexpression alone, the combined experimental group had decreased cell viability, increased iron content and ROS levels, and reduced GSH levels (*p* < 0.05, Figs. 4G-J). These results indicate that LINC00958 inhibits ferroptosis by binding to SRSF1 and increasing GPX4 expression.

### LINC00958 inhibits ferroptosis by binding to SRSF1 and increasing GPX4 expression

**Fig. 5:**
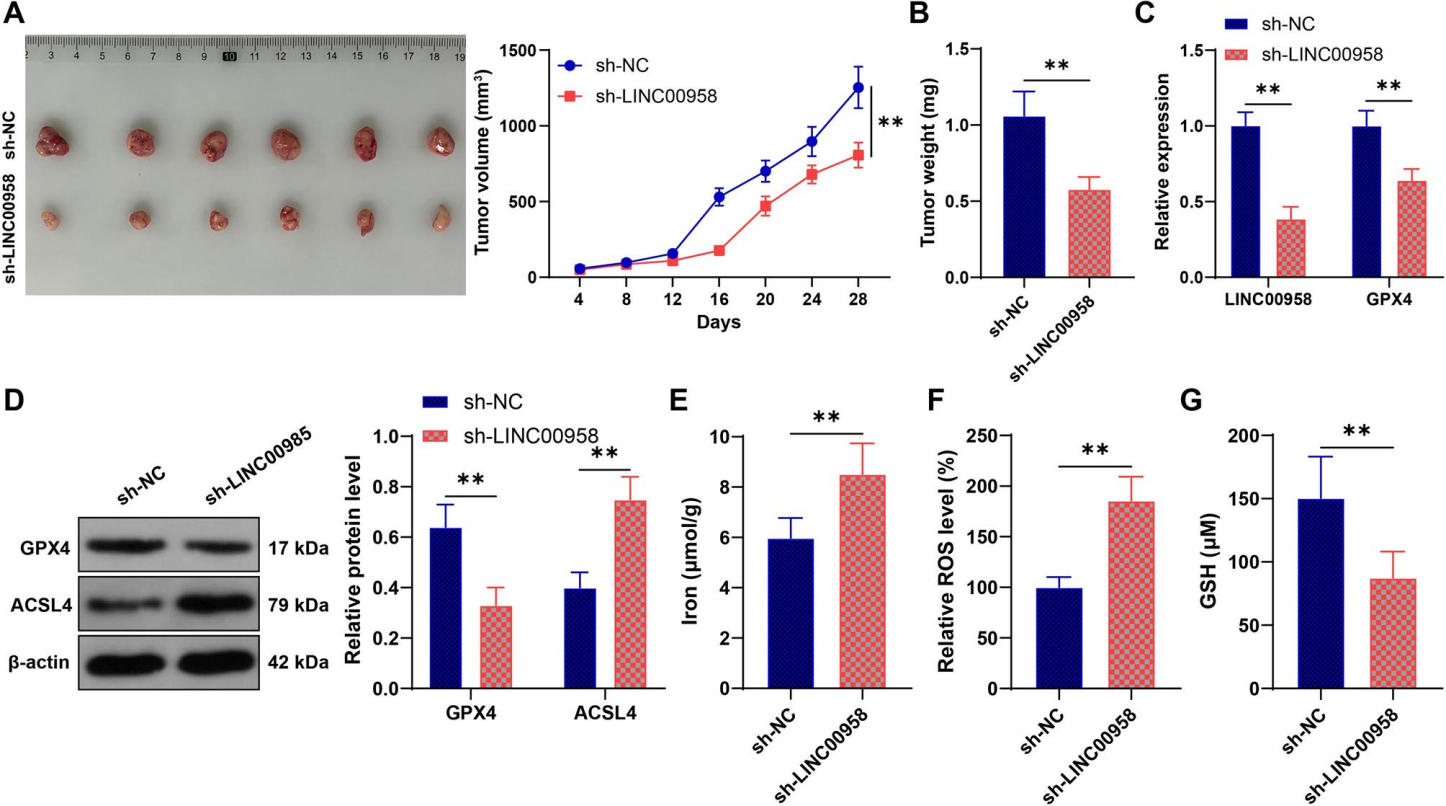
LINC00958 inhibits ferroptosis by binding to SRSF1 and increasing GPX4 expression. **A** xenograft model was constructed using MDA-MB-231 cells infected with lentivirus containing LINC00958 shRNA (sh-LINC00958), with MDA-MB-231 cells infected with sh-NC as the negative control. **A**: Tumor volumes were measured every four days, and representative images were photographed after euthanizing the nude mice on day 28. **B**: Tumor weights. **C**: RT-qPCR was used to detect the expression of LINC00958 and GPX4 in the cells. **D**: Western blot was used to detect the expression of GPX4 and ACSL4. **E**: Iron content in the tissues. **F**: ROS levels in the tissues. **G**: GSH levels in the tissues. *N* = 6. Experiments were independently repeated three times, and data are presented as mean ± standard deviation. Comparisons between two groups in panels **B**, **E**-**G** were performed using *t*-test, while comparisons among multiple groups in panels **A**, **C**-**D** were performed using two-way ANOVA, followed by Tukey’s multiple comparisons test. ** *p* < 0.01

Finally, we established a xenograft model using MDA-MB-231 cells infected with sh-LINC00958 and found that silencing LINC00958 led to a reduction in tumor volume and body weight (*p* < 0.05, Figs. 5A-B). Compared to the sh-NC group, the sh-LINC00958 group showed decreased LINC00958 expression and GPX4 expression in the tumor tissue (*p* < 0.05, Figs. 5C-D), along with elevated iron content, ROS levels, and ACSL4 expression, and reduced GSH levels (*p* < 0.05, Figs. 5D-G). In conclusion, LINC00958 inhibits ferroptosis in BC cells by binding to SRSF1 and increasing GPX4 expression.

## Discussion

**Fig. 6:**
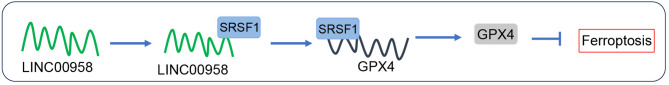
The LINC00958/GPX4 axis inhibits ferroptosis in BC cells. LINC00958 directly binds to SRSF1, leading to SRSF1 occupying more of GPX4, promoting GPX4 expression, and inhibiting ferroptosis in BC cells

Induction of ferroptosis in BC cells is considered a promising therapeutic approach that can effectively inhibit BC growth [28]. Notably, the regulatory connection between lncRNAs and ferroptosis is an effective therapeutic target for female-specific tumors, including BC [29]. Herein, we reported for the first time that LINC00958 directly bound to SRSF1 and augmented occupancy of SRSF1 on GPX4 mRNA, enhancing GPX4 mRNA stability, and subsequently promoting GPX4 expression, which ultimately inhibited ferroptosis in BC cells (Fig. 6). Our results may offer direction for developing new pathways for treating BC through LINC00958.

A large number of studies have shown that overexpressed lncRNAs may promote BC progression by inhibiting ferroptosis, for example, lncRNA DSCAM-AS1 [30], lncRNA PTPRG-AS1 [31], and lncRNA HCP5 [32]. In this study, we found that LINC00958 was overexpressed in BC and inhibited cellular ferroptosis, manifested by suppression of ACSL4 and increased expression of GPX4 in BC cells. A previous study has shown that downregulating LINC00958 reduces the survival, migratory distance, and invasive capability of bladder cancer cells [33]. Silencing LINC00958 inhibits cell viability, alleviates cell migration and invasion, and significantly enhances cell apoptosis of colorectal cancer by activating the PI3K/AKT pathway [34]. Importantly, highly expressed LINC00958 indicates a poor prognosis for BC patients and promotes tumor progression in BC cells [13]. These studies confirm the role of LINC00958 downregulation in driving tumor cell death. Consistently, after inhibiting LINC00958, BC cell viability was inhibited, and the iron content and ROS levels increased while GSH levels decreased, with elevated iron death-related protein ACSL4 expression and lower GPX4 expression in BC cells. In summary, our research has newly discovered that LINC00958 has the potential to promote ferroptosis and inhibit BC progression. For clinical translation, developing antisense oligonucleotides or siRNAs that target LINC00958 to specifically inhibit its expression, thereby promoting ferroptosis in BC cells, represents a promising therapeutic approach [35]. Nucleic acid-based therapies can be chemically modified to enhance stability and delivery efficiency, and they may be formulated into nanoparticles or liposomes for targeted delivery to BC tissues, reducing off-target effects [36].

Targeting GPX4 is considered a promising therapeutic strategy for inducing ferroptosis to treat cancer, as GPX4 can effectively inhibit lipid oxidation [37]. SRSF1 is a prototypical member of the serine/arginine-rich RNA-binding protein family and plays a crucial role in mRNA transcription, splicing, and translation [38]. Reports indicated that lncRNAs participate in cancer cell proliferation, invasion, and apoptosis by binding to SRSF1 [39, 40]. Small molecule inhibitors targeting SRSF1 have already demonstrated the effect of enhancing the anti-tumor immune response in animal models [41]. However, there is currently no evidence pointing to the specific mechanism by which LINC00958/SRSF1/GPX4 is involved in BC. Herein, our experimental data showed that LINC00958 interacted with SRSF1, altering the binding of SRSF1 to GPX4 mRNA and increasing the stability of GPX4 mRNA. Overexpression of GPX4 inhibited ferroptosis in BC cells in vitro. Another study reported that knocking down GPX4 induced total ROS accumulation and increased Fe^2+^ intensity in triple-negative BC cells, promoting ferroptosis [42]. GPX4 expression is inhibited in Escin-induced ferroptosis of BC cells [27]. Inhibiting GPX4 enzyme activity to promote ferroptosis can reduce radiotherapy resistance in BC cells [43]. Taken together, LINC00958 inhibits ferroptosis in BC cells by increasing GPX4 expression through SRSF1 both in vitro and in vivo.

Our study still has some limitations. First, as an initial exploration, the mechanisms we identified are somewhat simple. Second, it remains unclear whether there are other pathways through which LINC00958 regulates ferroptosis in BC cells. Third, the possibility that LINC00958 functions as a lncRNA via a ceRNA mechanism has not been investigated. Fourth, other biological behaviors, such as invasion and migration, were not investigated due to our funding limitations. Finally, the application of LINC00958 needs to be translated into clinical practice, such as nucleic acid-based therapies and small molecule inhibitors. In the future, we plan to further explore the downstream of LINC00958, incorporate clinical samples, and examine the regulatory impacts of LINC00958 on malignant behaviors such as apoptosis, migration, and proliferation in BC cells, providing new theoretical knowledge for the treatment of BC.

In conclusion, LINC00958 binds to SRSF1, leading to increased occupancy of SRSF1 on GPX4 mRNA, enhancing the stability of GPX4 mRNA, promoting GPX4 expression, and inhibiting ferroptosis in BC cells.

## Electronic supplementary material

Below is the link to the electronic supplementary material.

Supplementary Material 1

Supplementary Material 2

Supplementary Material 3

## Data Availability

The analyzed data sets generated during the present study are available from the corresponding author on reasonable request.

## Declarations

### Animal studies

Animal studies were performed in compliance with the ARRIVE guidelines. All procedures involving the use, care, and handling of animals were complied with the National Institutes of Health Guide for the Care and Use of Laboratory Animals [21].
