# Supplementary material for: Mechanism of LINC00958 in ferroptosis of breast cancer through the SRSF1/GPX4 axis
Source: Hereditas. 2025 Jun 19;162:110. doi: 10.1186/s41065-025-00469-6 (PMC12180173; doi:10.1186/s41065-025-00469-6)

Figure 1J
MDA-MB-157

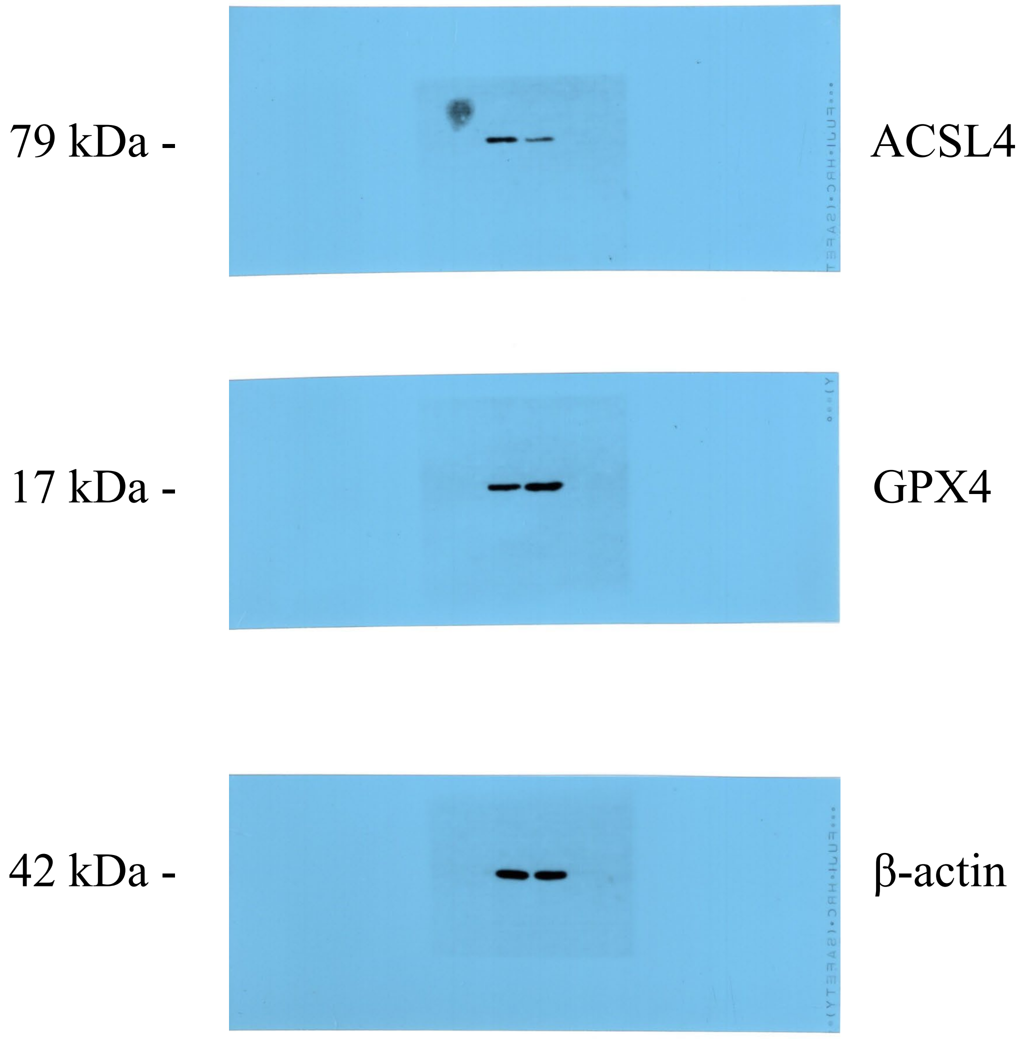

Figure 1J
MDA-MB-231

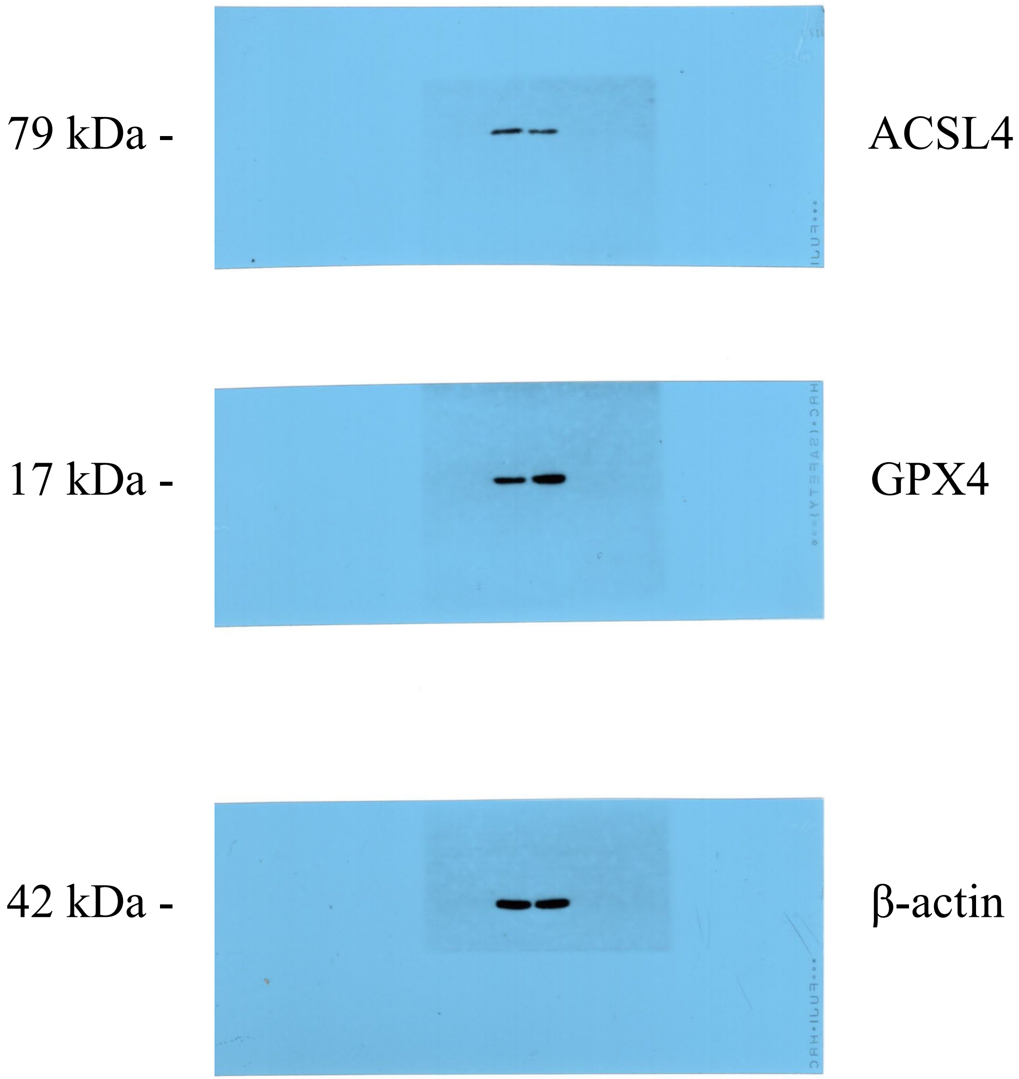

Figure 2F
MDA-MB-157

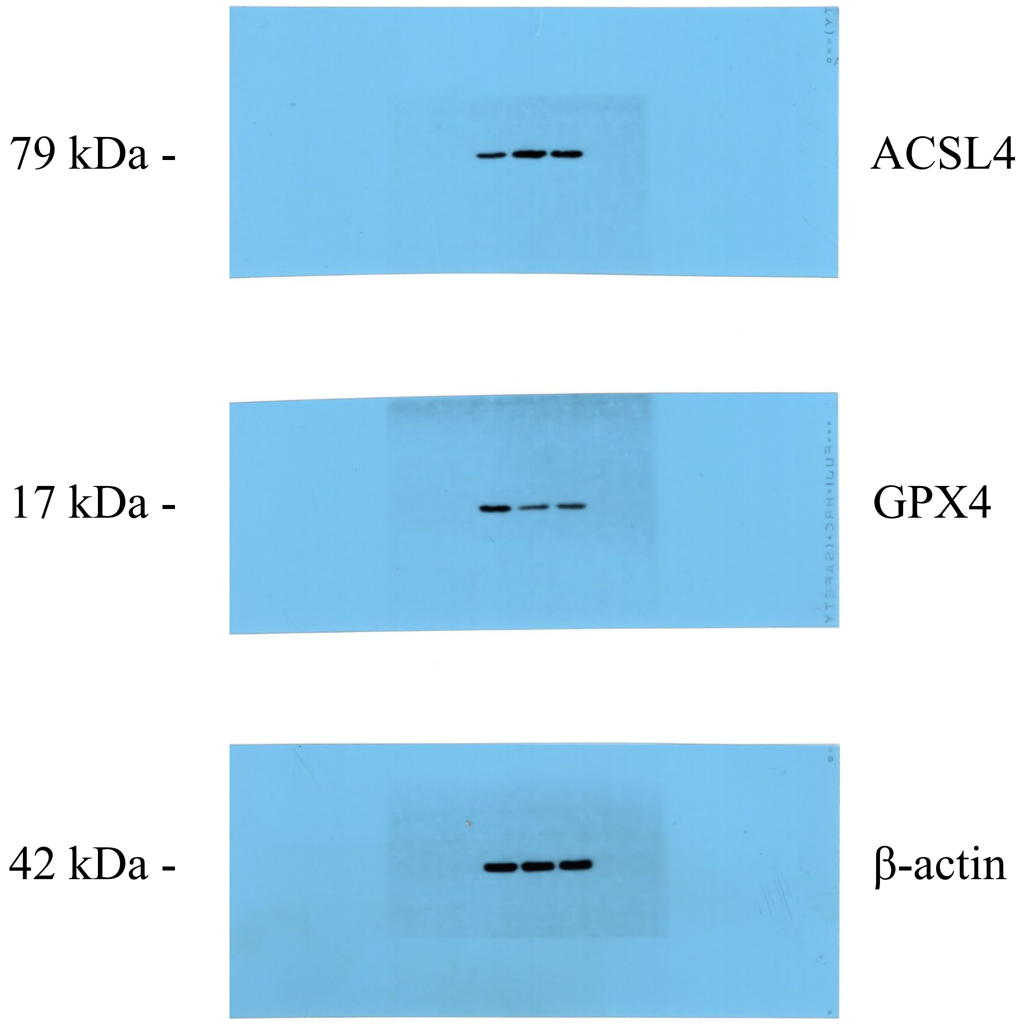

Figure 2F
MDA-MB-231

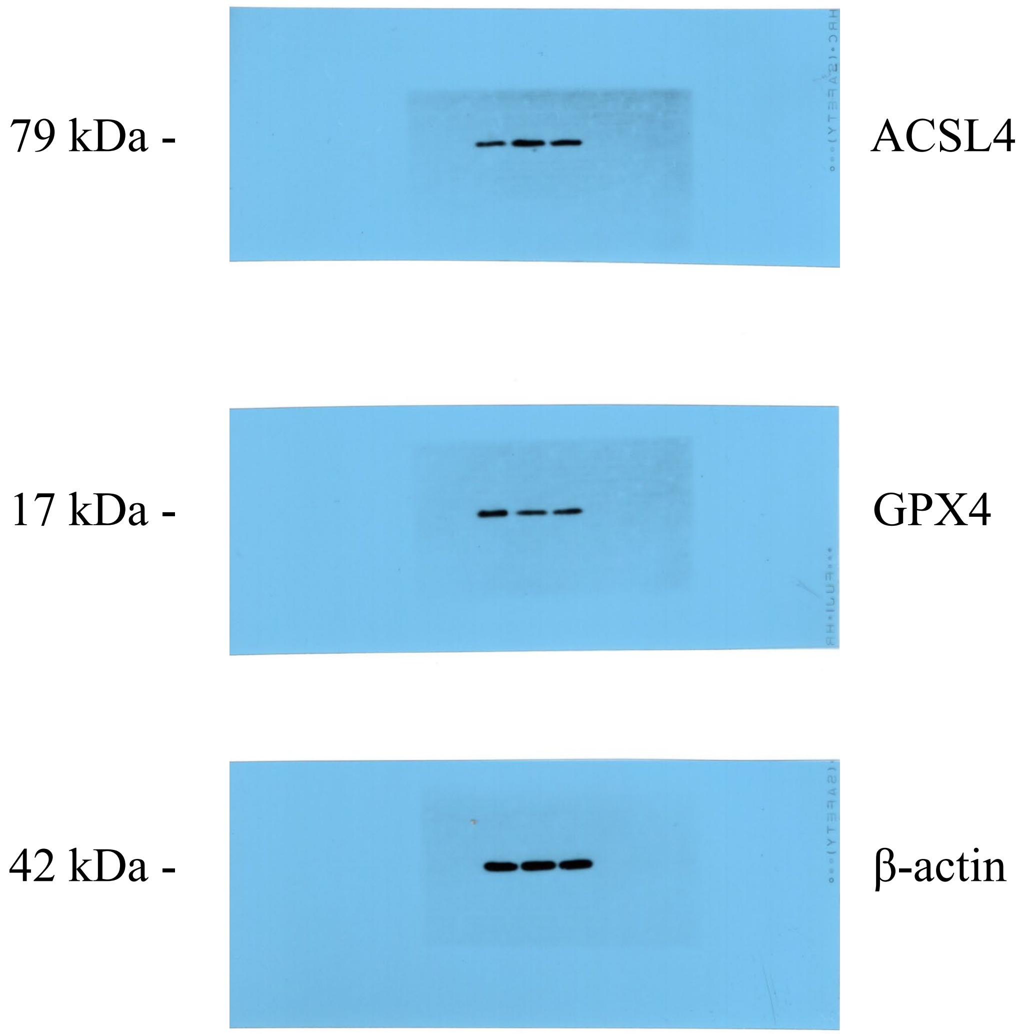

Figure 3F
SRSF1

27 kDa -
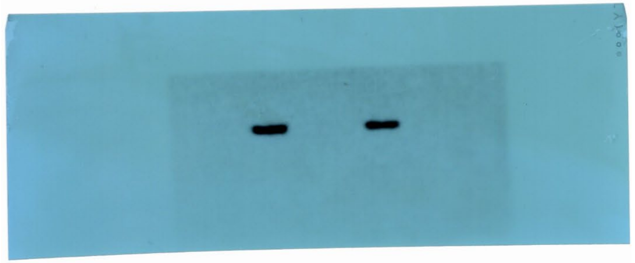
MDA-MB-157

27 kDa -
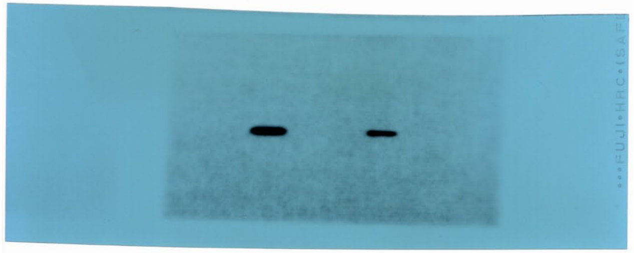
MDA-MB-231

Figure 3J
MDA-MB-157

27 kDa -

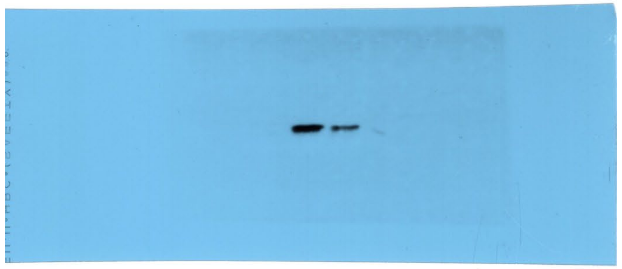

SRSF1

42 kDa -

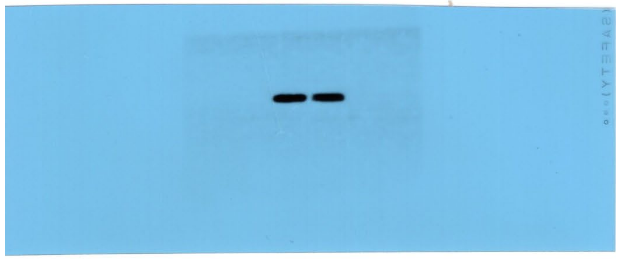

β-actin

Figure 3J
MDA-MB-231

27 kDa -
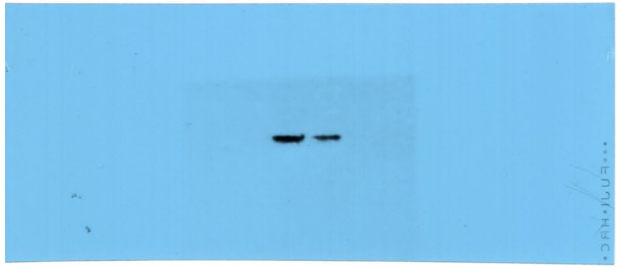
SRSF1

42 kDa -
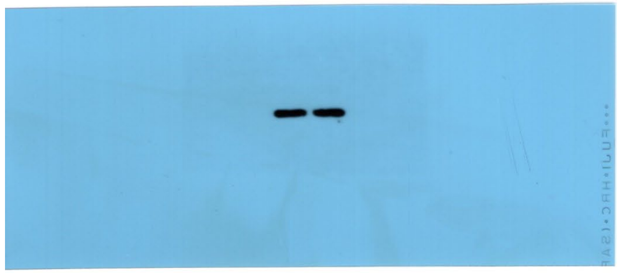
β-actin

Figure 3K

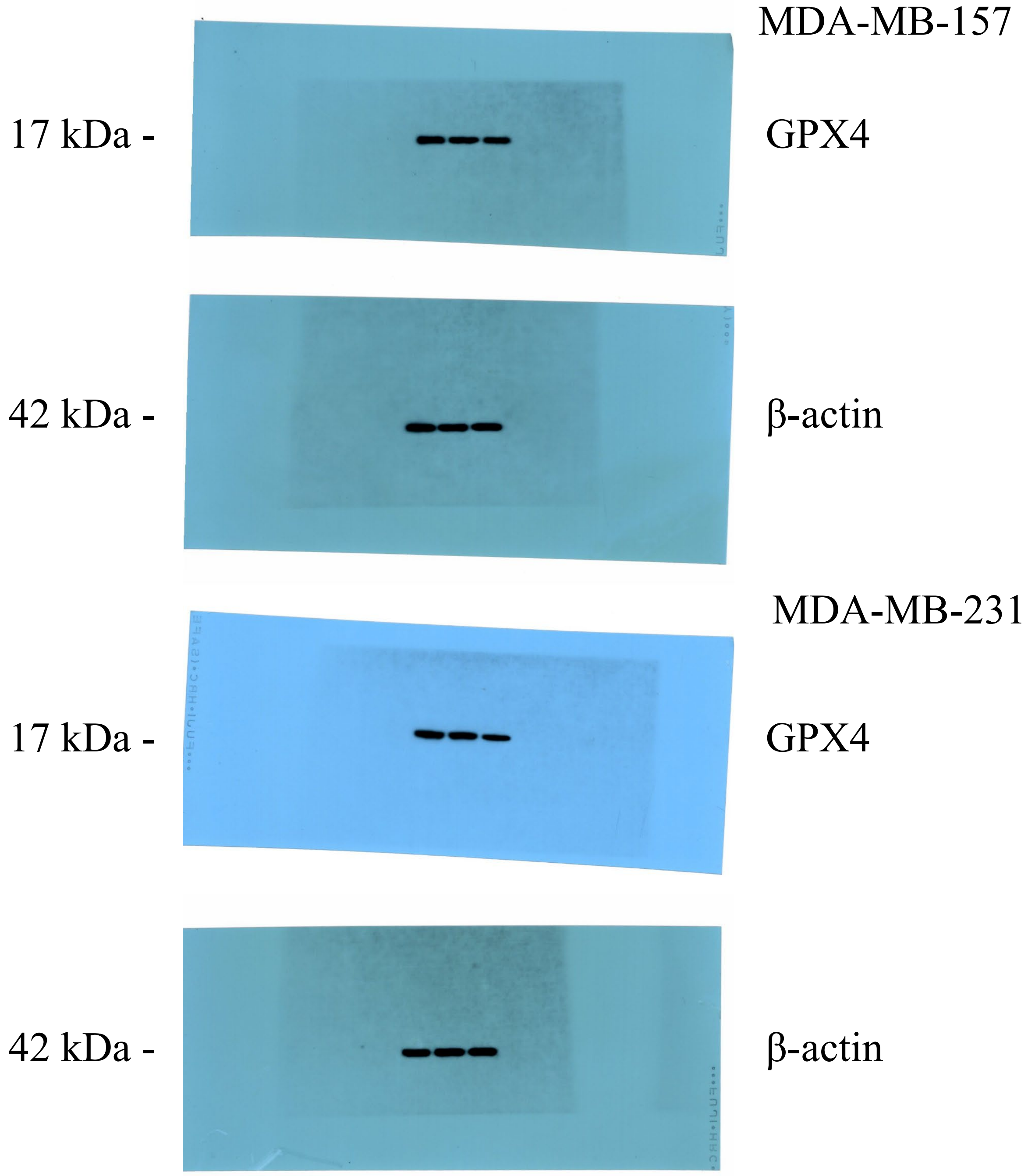
MDA-MB-157
17 kDa -
GPX4
42 kDa -
β-actin
MDA-MB-231
17 kDa -
GPX4
42 kDa -
β-actin

Figure 4C

MDA-MB-231

17 kDa - GPX4

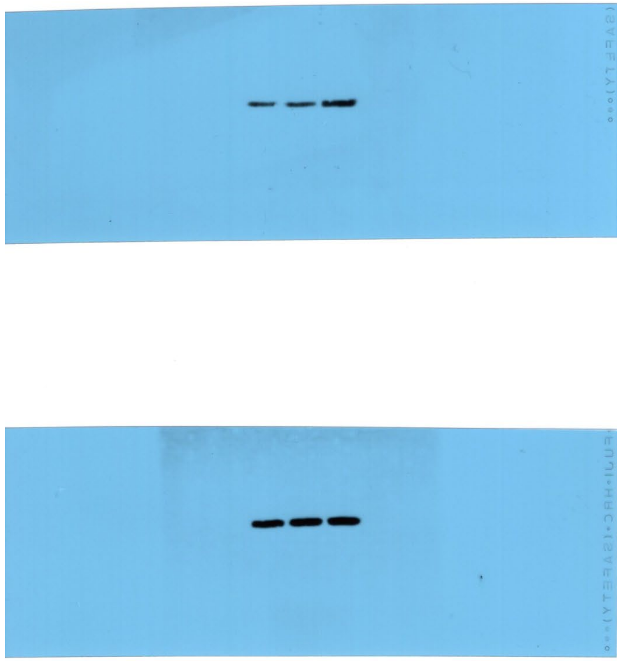

42 kDa - β-actin

Figure 4F
MDA-MB-157

17 kDa -
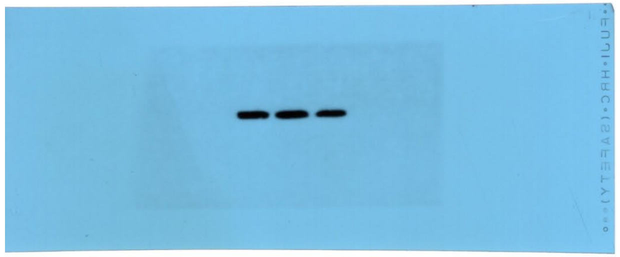
GPX4

42 kDa -
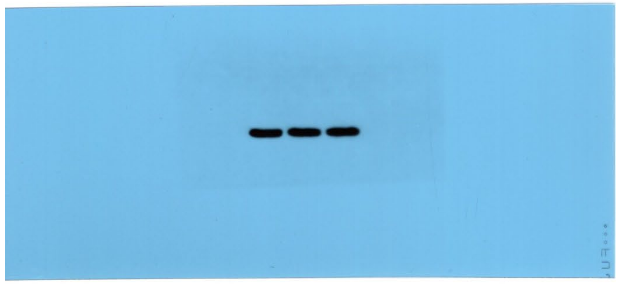
β-actin

Figure 5D

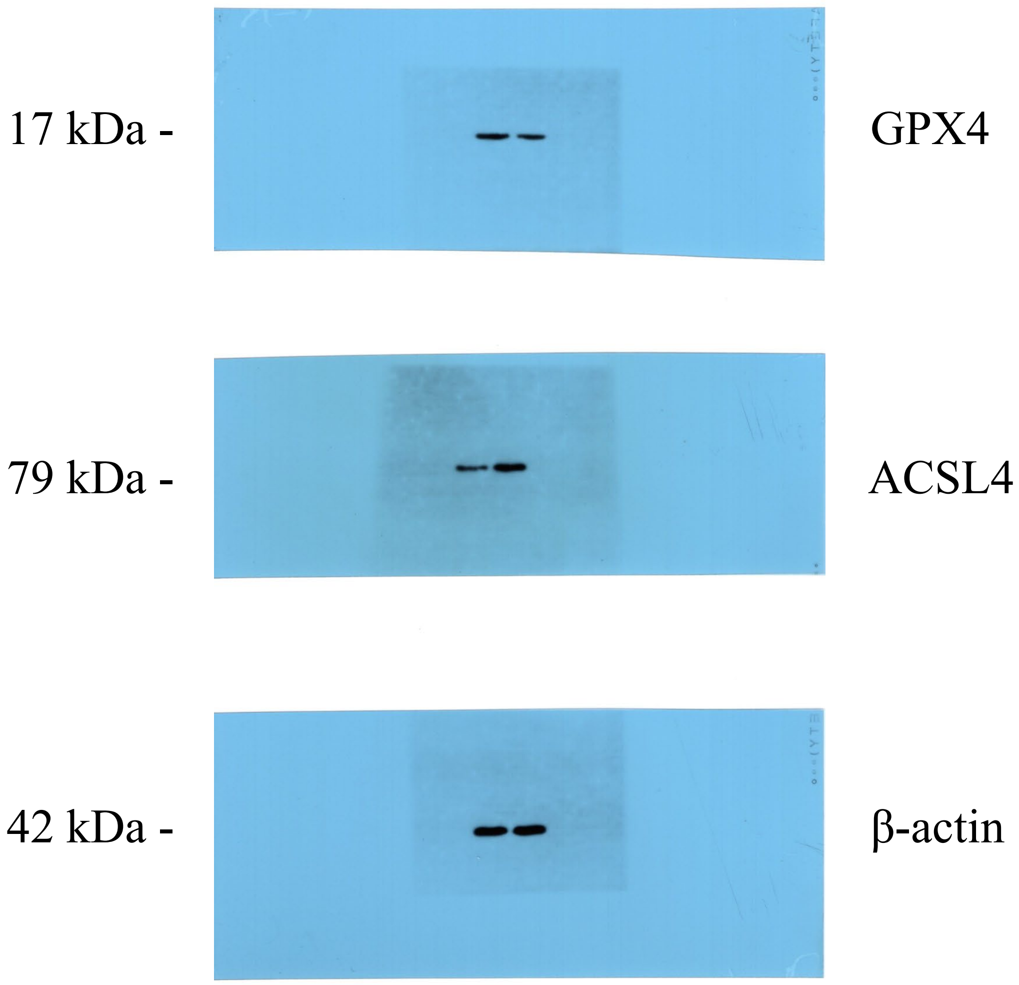

Supplement: Supplementary file 1 — Supplementary Material 1 [file 41065_2025_469_MOESM1_ESM.pdf]
