# Supplementary material for: Mechanism of LINC00958 in ferroptosis of breast cancer through the SRSF1/GPX4 axis
Source: Hereditas. 2025 Jun 19;162:110. doi: 10.1186/s41065-025-00469-6 (PMC12180173; doi:10.1186/s41065-025-00469-6)
Supplement: Supplementary file 2 — Supplementary Material 2 [file 41065_2025_469_MOESM2_ESM.docx]

**Supplementary Table 1** siRNA sequences

| Gene | Sequences (5’-3’) |
| --- | --- |
| si-LINC00958-1 | SS: GGUUAAGACAUUUCUACAAAU |
|  | AS: UUGUAGAAAUGUCUUAACCUG |
| si-LINC00958-2 | SS: GAGUGGAGAGACUCAGCUACC |
|  | AS: UAGCUGAGUCUCUCCACUCAG |
| si-LINC00958-3 | SS: GCAGGUUAAGACAUUUCUACA |
|  | AS: UAGAAAUGUCUUAACCUGCAA |
| si-SRSF1-1 | SS: GGUAAGUAUUAAAUGUUAACA |
|  | AS: UUAACAUUUAAUACUUACCUU |
| si-SRSF1-2 | SS: GAAGAUAGAUGAAAGCUUAGA |
|  | AS: UAAGCUUUCAUCUAUCUUCAG |
| si-GPX4-1 | SS: CACAGUUCCUCAUCGACAAGA |
|  | AS: UUGUCGAUGAGGAACUGUGGA |
| si-GPX4-2 | SS: GGAGUAACGAAGAGAUCAAAG |
|  | AS: UUGAUCUCUUCGUUACUCCCU |
